# Two New Species of *Primula* Sect. *Auganthus* From Sichuan, China

**DOI:** 10.1002/ece3.72047

**Published:** 2025-08-27

**Authors:** Si‐Yu Zhang, Ying‐Feng Hu, Jiu‐Lin Gu, Yang Hu, Xiao‐Yu Si, Wei Zhang, Jian‐Wen Shao

**Affiliations:** ^1^ College of Life Sciences Anhui Normal University Wuhu Anhui China; ^2^ College of Civil and Architecture Engineering Chuzhou University Chuzhou Anhui China; ^3^ College of Pharmacy Guizhou University of Traditional Chinese Medicine Guiyang Guizhou China; ^4^ Chengdu Shanhualanmanshi Gardening Limited Company Chengdu Sichuan China; ^5^ College of Life Sciences Anqing Normal University Anqing Anhui China; ^6^ The Anhui Provincial Key Laboratory of Biodiversity Conservation and Ecological Security in the Yangtze River Basin Anhui Normal University Wuhu Anhui China

**Keywords:** new species, phylogeny, *Primula*, sect. *Auganthus*, species delimitation

## Abstract

In groups undergoing rapid radiations, species delimitation among phylogenetically close sister lineages has long been a challenge. During plant surveys in northwestern Sichuan, we unexpectedly discovered two putative new species of *Primula* that are morphologically similar yet distinct from each other. These species resemble *P. xingshanensis*, which has been assigned to sect. *Auganthus* based on morphological characters. To clarify the precise phylogenetic positions of the two putative new species and *P. xingshanensis*, we sampled related taxa and conducted phylogenetic analyses using chloroplast genomes and nuclear SNPs. The results showed that the two putative new species form sister clades and are closely related to *P. sinensis* , while *P. xingshanensis* is sister to *P. rupestris* . All belong to the sect. *Auganthus*. Based on population genetic structure, morphological statistics, and artificial hybridization experiments, both putative new species should be accepted as distinct species, herein formally described as *P. rongrong* sp. nov. and *P. fujiangensis* sp. nov. Based on our field surveys and in accordance with the IUCN criteria, we assess the conservation status of *P. rongrong* as least concern (LC) and *P. fujiangensis* as critically endangered (CR).

## Graphical abstract

We describe two new sister species, *Primula rongrong* and *P. fujiangensis* (sect. *Auganthus*), from Sichuan, China. Phylogenomic analyses (chloroplast + nuclear SNPs) confirm their sister relationship within the section. Significant morphological divergence (PCA of 16 traits; **p < 0.05 for 8 characters) and reproductive isolation (13.75% interspecific vs. 67.5%–80% intraspecific fruit set) validate their distinct species status.

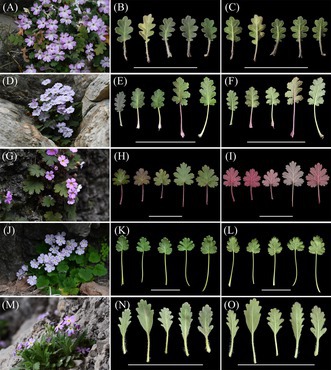

## 1 Introduction

Species are the basic units of biodiversity and the foundation upon which all life science research relies. The delineation of species has always been a focal point for biologists, as it is crucial for biodiversity protection and resource utilization. With technological advancements, systematic research based on molecular biology has been widely applied in species delineation. It can, to some extent, reflect the phylogenetic relationships and evolutionary order among species and can also delineate species through means such as genetic distance. However, the concept of species remains relatively vague, with many species still on a rapid evolutionary path (Mayr 1942; de Queiroz 2007; Sites and Marshall 2003). Relying solely on molecular methods for species distinction may lead to overly fine delineations and conclusions that do not align with objective reality (Carstens et al. 2013). Morphology plays a significant role in traditional taxonomy, but classifying solely based on morphology makes it difficult to avoid the pitfalls brought by convergent evolution of superficial traits, which may result in erroneous species classification or even misplacement of various branches within the classification system (Wiens 2004; Wake et al. 2011). In addition, evidence from reproductive isolation (IR) also proves valuable in resolving taxonomic issues within complex groups, especially herbaceous groups with narrow and disjunct distributions (Hu et al. 2018; He et al. 2022). Therefore, the current “integrated classification method” based on multiple evidences, such as molecular systematics, morphology, and IR is considered to be able to obtain relatively reasonable classification conclusions and is an effective means for species delineation (Dayrat 2005; Schlick‐Steiner et al. 2010).

*Primula* L. is a large genus widely distributed across various regions of the Northern Hemisphere, encompassing up to 548 species (POWO 2025). With as many as 300 species natively distributed in China, the Himalayan region and the mountainous areas of southwest China are currently recognized as the centers of diversity for this genus (Hu 1990; Hu and Kelso 1996). The combined effects of hybridization, evolutionary transitions between heterostylous and homostylous floral systems, and adaptive radiation present persistent challenges to the taxonomy of this genus (Mast et al. 2001; Ren et al. 2015; He et al. 2022; Zhang et al. 2023). Section *Auganthus* Pax ex Balf. f. is a small clade within *Primula*, primarily distributed in the mountainous areas surrounding the Sichuan Basin in China (Balfour 1913; Balfour and Farrer 1918; Hu 1990; Hu and Kelso 1996). Currently, molecular phylogenetic studies based on ITS sequences and chloroplast genome sequences support that three species are considered members of this section: *P. sinensis* Sabine ex Lindl., *P. filchnerae* R. Knuth, *P. rupestris* Pax & K. Hoffm. (Hao et al. 2002; Xu et al. 2020). Additionally, the newly described *P. xingshanensis* Yu B. Wang was placed in this section upon its publication, but molecular phylogenetic evidence supporting this placement is lacking (Wang et al. 2023).

**FIGURE 1:**
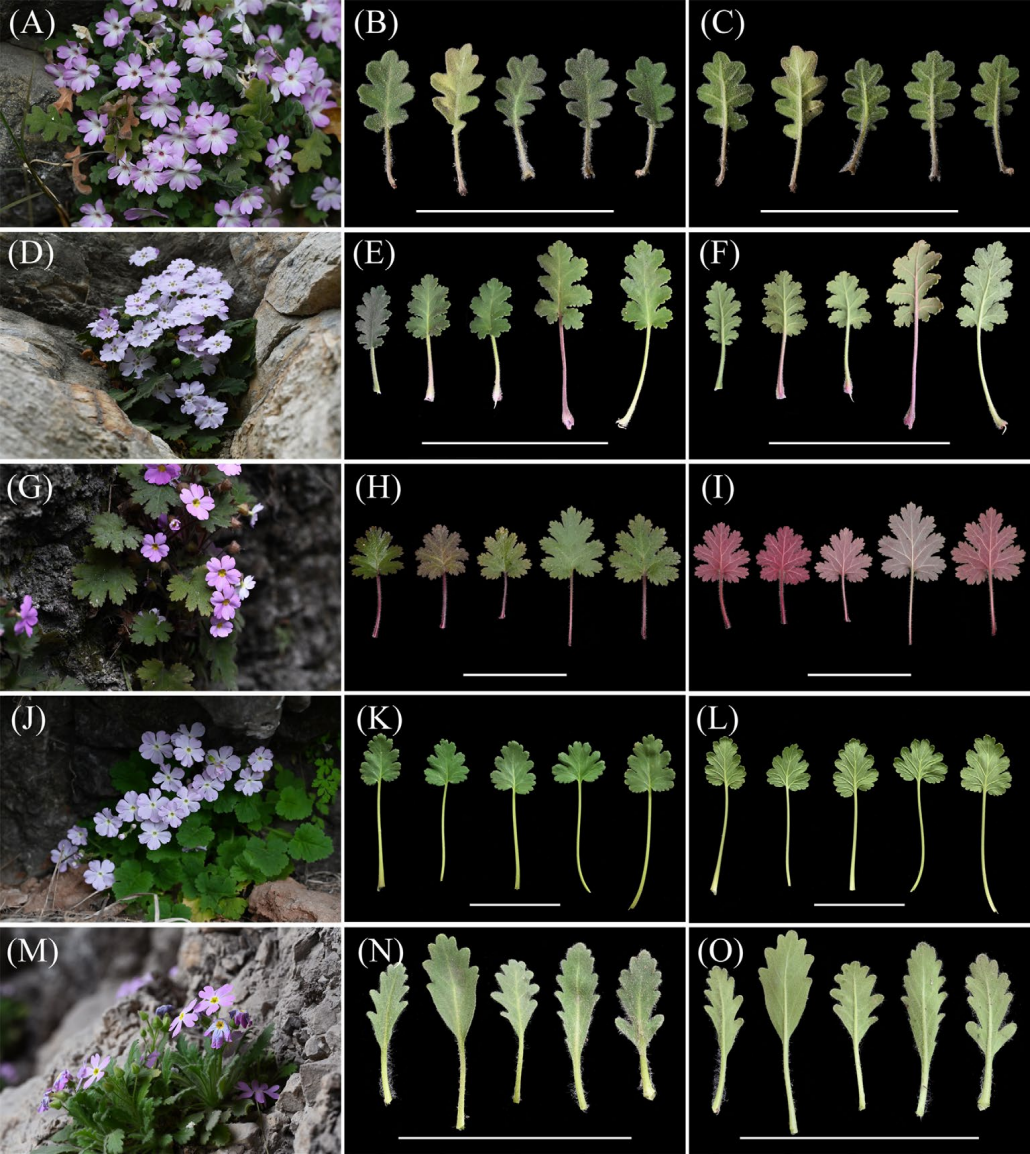
Morphological comparison between the new species and its close relatives. (A–C) *Primula rongrong* sp. nov (*P*. sp1); (D–F) *P. fujiangensis* sp. nov (*P*. sp2); (G, H) *P. sinensis* ; (J–L) *P. rupestris* ; (M–O) *P. xingshanensis*. The first vertical column shows plant photos, the second vertical column shows the upper surface of leaves, the third vertical column shows the lower surface of leaves, and the scale length is 10 cm.

In spring 2024, during a wild plant survey in the eastern edge of Northwestern Sichuan Province, we discovered a suspected new taxon of *Primula* (*P*. sp1). Based on its hairy pinnatifid leaves, it is close to *P. xingshanensis* and thus likely belongs to sect. *Auganthus* (Figure 1). To understand the accurate distribution of this putative new species, we expanded our survey scope but found no additional populations. However, on another cliff 50 km away, we discovered another suspected new taxon of *Primula* (*P*. sp2) morphologically similar to but distinct from *P*. sp1. The two putative new species differ in indumentum, leaf lobes, and nectar guides, yet exhibit consistency in overall plant morphology (e.g., plant height) and habit. These discoveries left us puzzled: What is the relationship between these two putative taxa? Are they one or two distinct new taxa? Do they belong to the sect. *Auganthus*? Since both putative new species have only one population, obtaining more samples to enhance research credibility is difficult, posing challenges in answering these questions and risking inaccurate taxonomic treatment. Reviewing relevant taxonomic data, we found that the distance between these two putative species and the only known population of morphologically similar *P. xingshanensis* exceeds 500 km, showing highly discontinuous distribution. Thus, is *P. xingshanensis* phylogenetically close to these two putative species? To resolve the aforementioned questions and further elucidate the phylogenetic relationships within sect. *Auganthus*, we conducted field investigations and collected samples of plants in this section. We propose to carry out research by integrating evidence from molecular phylogenetic analyses based on genome‐wide single nucleotide polymorphisms (SNPs) and chloroplast genome sequences, morphometric statistics, and artificial crossing experiments.

## 2 Material and Methods

### 2.1 Sample Collection and Statistical Analysis of Morphology

Field surveys and sampling were conducted for two putative new species, along with *Primula sinensis* , *P. rupestris* , and *P. xingshanensis* belonging to sect. *Auganthus*. *P. filchnerae* was excluded from sampling due to its rarity and the availability of three complete chloroplast genome sequences in public databases (Hu 1990; Hu and Kelso 1996).

**FIGURE 2:**
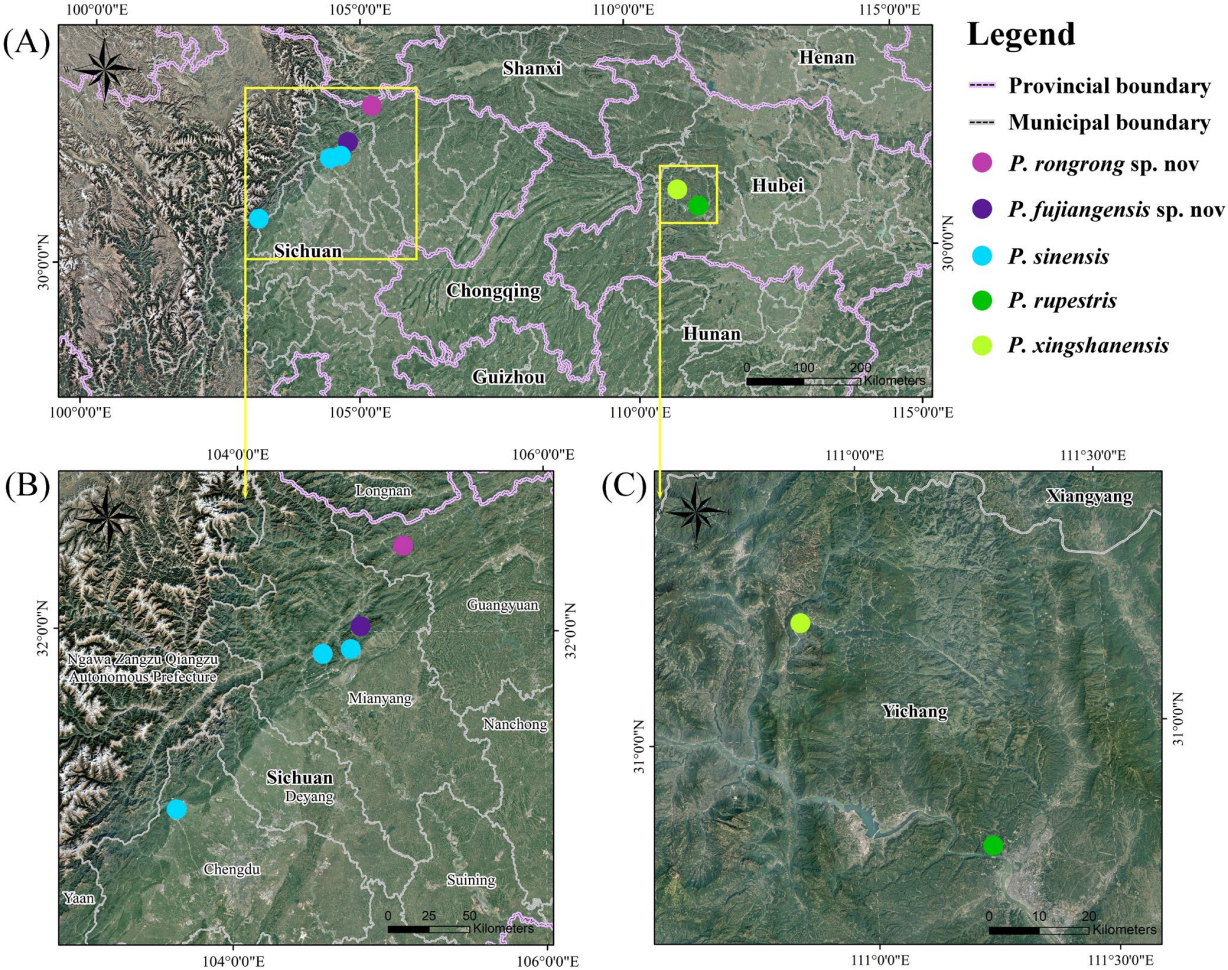
Distribution map of sampling sites for *Primula* sect. *Auganthus*. (A) Distribution of all sampling sites; (B) Sampling locations in northwestern Sichuan Province; (C) Sampling locations in western Hubei Province.

Sampling was carried out from February to March 2024. Fresh leaves were collected in the field and dried using silica gel for DNA sequencing. Concurrently, a large number of photographs were taken, and a small number of specimens were collected for morphological comparison between the new species and its closely related species, for the preparation of type specimens and the description of the new species. The locations of the wild populations surveyed, the sampling information for DNA sequencing, and the specimen numbers for some voucher‐collected samples are listed in Table 1. Voucher specimens are deposited in the Herbarium, College of Life Sciences, Anhui Normal University (ANUB). The distribution map of the sampling locations is shown in Figure 2.

**TABLE 1:** Sampling information of plants in *Primula* sect. *Auganthus*.

Locations	Code	GenBank acc. no	Voucher specimens
*P. rongrong* sp. nov			
Fangshi Town, Qingchuan County, Guangyuan City, Sichuan Province	01	PV839561	ANUB100111
	02	PV839562	ANUB100112
	03	PV839563	ANUB100113
*P. fujiangensis* sp. nov			
Yongsheng Town, Jiangyou City, Mianyang City, Sichuan Province	01	PV839556	ANUB100115
	02	PV839557	ANUB100116
*P. sinensis*			
Longchi Town, Dujiangyan City, Chengdu City, Sichuan Province	DJY 01	PV839558	ANUB100117
	DJY 02	PV839559	ANUB100118
	DJY 03	PV839560	ANUB100119
Hanzeng Town, Jiangyou City, Mianyang City, Sichuan Province	QYS 01	PV839549	/
	QYS 02	PV839550	/
Qushan Town, Beichuan County, Mianyang City, Sichuan Province	bc 01	PV839547	/
	bc 02	PV839548	/
*P. rupestris*			
Xiaoxita Subdistrict, Yiling District, Yichang City, Hubei Province	01	PV839553	ANUB100120
	02	PV839554	ANUB100121
	03	PV839555	ANUB100122
*P. xingshanensis*			
Xiakou Town, Xingshan County, Yichang City Hubei Province	01	PV839551	ANUB100123
	02	PV839552	ANUB100124

To compare the morphological differences between the two putative new species, morphological statistics were performed. For population protection purposes, we did not collect a large number of specimens. Instead, 30 flowering individuals with an interval of more than 2 m between individuals in the population were randomly selected for morphology observation in the wild. A moderately sized leaf was picked from each plant, photographed on a black light‐absorbing cloth with a scale, and morphologically measured and counted using ImageJ v. 1.54d (Schneider et al. 2012) indoors. A total of 10 leaf morphological indices were statistically analyzed: leaf area, leaf perimeter, leaf shape index (leaf perimeter/leaf area), leaf length (excluding petiole), leaf width, petiole length, petiole ratio [petiole length/(leaf length + petiole length)], primary lobes number, total serration number on lobes, and lateral veins number. Six flower‐related morphological indices were measured on‐site using a vernier caliper in the field. For each individual, a moderately sized inflorescence was selected to measure the inflorescence height and the length of a random bract. The presence of nectar guides (absent: 0, present: 1) and the color of the flower throat (white: 0, purple: 1) were observed. A small flower with a moderate pedicel length was selected from each inflorescence to measure the pedicel length and calyx tube length.

All data were entered into Microsoft Office v. 2021 for calculation and collation. Principal component analysis (PCA) was performed on all 16 morphological indices, 10‐leaf morphological indices, and 6‐flower morphological indices using Origin v. 2021. Origin v. 2021 was also used to visualize all measurement and count indices, with measurement indices presented as boxplot + normal curve plots and count indices as violin + box plots. SPSS v. 25 was used for significance analysis of differences for all measurement and count indices. First, the Shapiro–Wilk test was used to check whether the data conformed to a normal distribution. For indices that conformed to a normal distribution (leaf area, total serration number on lobes, and lateral vein number), independent samples *T*‐tests were performed, and other indices were tested using the Mann–Whitney *U* test for nonparametric data. The final results were presented using the significance notation method.

### 2.2 DNA Extraction, Sequencing, and Phylogenetic Reconstruction

DNA samples were extracted from leaves dried by silica gel according to the modified cetyltrimethylammonium bromide (mCTAB) extraction protocol (Doyle and Doyle 1987; Larridon et al. 2015). Extracted DNA quality was verified by NanoDrop 1000 spectrophotometry and 0.8% agarose gel electrophoresis. Library construction and 150‐bp paired‐end sequencing were outsourced to Frasergen Biotechnology Co. Ltd. (Wuhan, China) using Illumina HiSeq 6000, with libraries prepared from 1 μg DNA/sample via VAHTS Universal Plus DNA Library Prep Kit for Illumina (Vazyme), incorporating dual 8‐bp indexes (i7 + i5, TruSeq CD Set, Hamming distance ≥ 3). Frasergen performed library quality control (QC) with the following criteria: Qubit concentration ≥ 20 nM; Bioanalyzer results showing a peak at 450 ± 50 bp and adapter dimer content < 5%. Approximately 3 Gb of raw data per sample were generated and utilized for chloroplast genome assembly and annotation, as well as nuclear SNP discovery.

**FIGURE 3:**
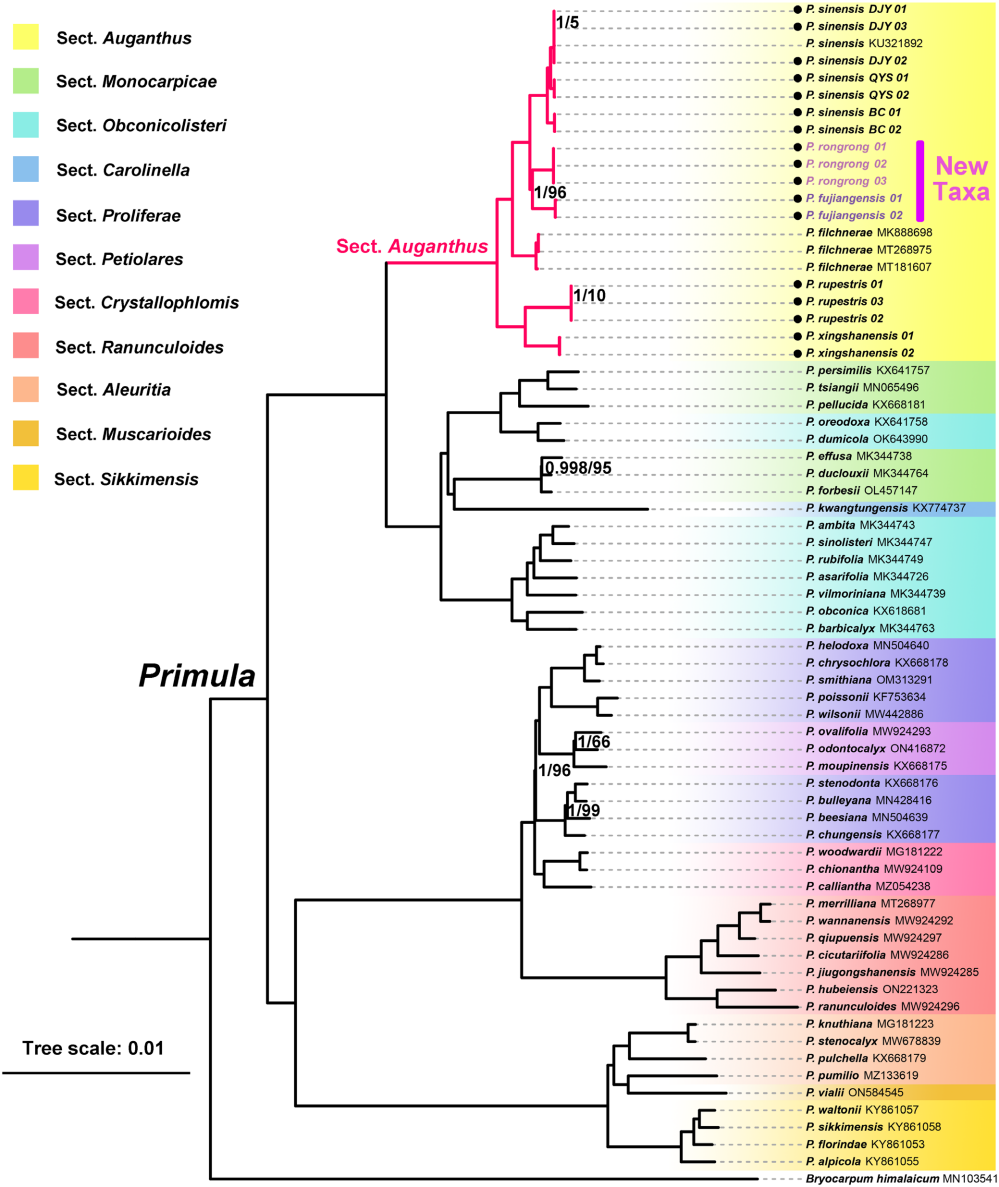
Phylogenetic tree of partial Chinese *Primula* species based on chloroplast genomes. Numbers above branches are Bayesian posterior probability (BPP)/maximum likelihood bootstrap values (BS); branches without labeled support values indicate 1/100. The species names with accession numbers were downloaded from NCBI, ● before species names denotes newly uploaded sequences in this study.

For chloroplast genome analysis, we used GetOrganelle v.1.7.1 to obtain the complete chloroplast genome, followed by genome annotation with PGA (Qu et al. 2019; Jin et al. 2020). This study was performed on the 17 newly reported complete chloroplast genomes (Table 1) and 51 complete chloroplast genomes of Chinese *Primula* that were downloaded from NCBI. *Bryocarpum himalaicum* was selected as the outgroup. See Figure 3 for the specific accession number of a total of 69 complete chloroplast genomes that were used for phylogenetic analysis.

For nuclear genomic analysis, raw sequencing reads were processed using SOAPnuke v.2.1.7 (Chen et al. 2018) to obtain clean reads. These clean reads were then mapped to the *Primula* *veris* reference genome (Nowak et al. 2015) using BWA v.0.7.17‐r1188 (Li and Durbin 2009) with default parameters. The resulting alignments were converted to BAM format, and duplicate reads were marked and removed using Picard tools (https://broadinstitute.github.io/picard/). Mapping statistics, including mapping rate, coverage, and depth for each sample, were calculated using SAMtools v.1.9 (Li et al. 2009). SNPs were called using GATK v.4.0 (DePristo et al. 2011). Finally, the initial SNP set was filtered using PLINK v.1.07 (Purcell et al. 2007) based on thresholds for missing rate (‐‐geno 0.2), minor allele frequency (‐‐maf 0.01), and linkage disequilibrium (LD, ‐‐indep‐pairwise 50 5 0.2); a total of 150,248 SNP loci were retained for phylogenetic analysis and population genetic structure analysis.

Phylogenetic trees were constructed separately based on the chloroplast genome sequence matrix and the nuclear SNP data matrix obtained from the procedures described above. All sequences were aligned by MACSE v.2 (Ranwez et al. 2018). Maximum likelihood (ML) and Bayesian inference (BI) methods were used to determine the phylogenetic relationships after the best‐fit model of DNA substitution being estimated by ModelFinder (Minh et al. 2013; Kalyaanamoorthy et al. 2017). ML analysis was conducted using the GTR + G + I model with 1000 bootstrap replicates by IQtree v.1.6.8 (Nguyen et al. 2015). Bayesian analysis was constructed with eight independent chains for 50,000,000 generations and sampled every 1000 generations by MrBayes v.3.2.6 (Ronquist et al. 2012). All phylogenetic analyses were performed in Phylosuite (Zhang et al. 2020).

Population genetic structure was assessed using Admixture (Alexander and Lange 2011) by testing *K* = 2–5 with five replicates per *K* and 10‐fold cross‐validation (CV) to determine the optimal cluster number and using Microsoft Office v. 2021 to visualize the Admixture results.

### 2.3 Artificial Pollination Experiments

Due to the adaptive constraints of plant growth, the artificial pollination experiment was conducted at Shan'hua'lan'man'shi Horticultural Farm in Chengdu, Sichuan Province, which is relatively close to the original habitat. Owing to limited experimental conditions, statistics could not be precise to the ovule level. The specific experimental design is as follows.

In June 2024, seeds were collected from the original habitats of two putative new species (*P. rongrong* = R, *P. fujiangensis* = F). For each species, 10 individuals were selected, with a minimum spacing of 3 m between individuals. One mature, plump seed capsule was collected from each individual. Capsules from the 10 individuals per species were pooled separately by taxon prior to transfer to the controlled greenhouse facility in Chengdu for sowin. In October 2024, seedlings were transplanted. In February 2025, for each species, five long‐styled (L) individuals and five short‐styled (S) individuals were selected for artificial pollination experiments. A total of eight combinations were set up: R L♂ × R S♀, R S♂ × R L♀, F L♂ × F S♀, F S♂ × F L♀, R L♂ × F S♀, R S♂ × F L♀, F L♂ × R S♀, and F S♂ × R L♀. For each pollination treatment, approximately 20 newly opened flowers were used. To prevent stigma contamination by self‐pollen, the flowers receiving pollen were emasculated before pollination. No more than three flowers per inflorescence and 10 flowers per plant were used for pollination, and no more than two flowers from the same plant were used in the same pollination treatment. In May 2025, the fruit‐setting status was recorded. With reference to the natural fruit‐setting status of flowers without artificial pollination, the fruit was considered to have developed normally if it swelled and more than five obviously swollen ovules were found upon dissection; otherwise, it was regarded as aborted.

## 3 Result and Discussion

### 3.1 Phylogeny Based on Chloroplast Genome

The phylogenetic tree of some *Primula* species distributed in China constructed based on chloroplast genomes is shown in Figure 3. Except for some terminal branches, most major clades received extremely high support values in both BI and ML analyses. We labeled the sections of these species referring to the infrageneric classification system in *Flora of China* (Hu 1990; Hu and Kelso 1996) and found that the phylogenetic positions of sect. *Monocarpicae*, sect. *Obconicolisteri*, and other sections, or the sectional affiliations of certain species, might still be controversial, which was also reported in Liu's (2018) investigation of the phylogeny of Chinese *Primula* species based on orthologous protein‐coding nuclear genes derived from transcriptome sequencing. However, from this phylogenetic tree, the monophyly of sect. *Auganthus* is unquestionable, and it forms a sister group relationship with the ancestral branches of sect. *Monocarpicae*, sect. *Obconicolisteri*, and sect. *Carolinella*.

The chloroplast phylogenetic relationships indicated that the two putative new species both belonged to sect. *Auganthus* and were sisters to each other. For *Primula sinensis* , although the DJY population was geographically distant from the other two populations, the three populations still formed a highly supported monophyletic group, which was sister to the ancestral branch of the two putative new species. Surprisingly, *P. xingshanensis*, which we considered morphologically close to the new species, was a sister species to *P. rupestris* and diverged basally from other members of sect. *Auganthus*. Overall, when observing the phylogenetic tree in combination with Figures 1, 2, the clade relationships within sect. *Auganthus* appear more closely associated with geographical distribution (*P. filchnerae* distributed in southern Shaanxi bridges the Sichuan and Hubei clades) rather than direct morphological relationships. The transition between palmate and pinnate lobes likely occurred multiple times in different clades.

### 3.2 Phylogeny and Population Genetic Structure Based on Nuclear Gene SNPs

**FIGURE 4:**
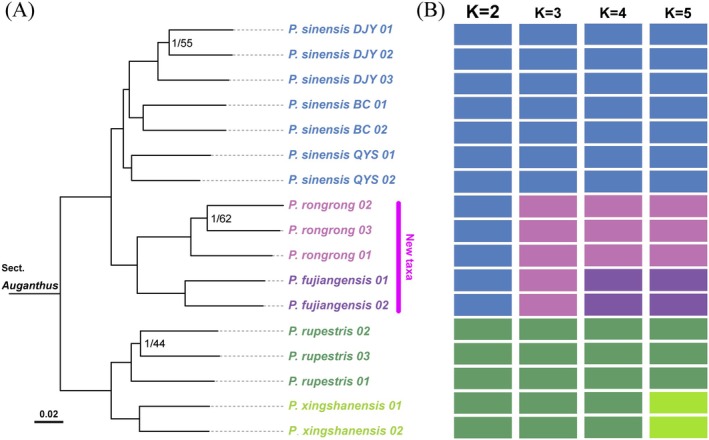
Phylogenetic tree and population genetic structure of *Primula* sect. *Auganthus* based on nuclear gene SNPs. (A) Phylogenetic tree, numbers above branches are Bayesian posterior probability (BPP)/maximum likelihood bootstrap values (BS), branches without labeled support values indicate 1/100; (B) Population genetic structure based on Admixture analysis, individuals correspond one‐to‐one with the phylogenetic tree on the left, with the bolded *K* value indicating the optimal model suggested by the program.

Based on the sampled populations of sect. *Auganthus* in this study, the phylogenetic tree from nuclear SNPs (Figure 4A) showed clade relationships consistent with the chloroplast tree. All branches received extremely high support values except for some within‐population subclades, and the two putative new species remained sisters. Significant genetic distances between the two putative new species exceeded those among other clades.

The Admixture results (Figure 4B) identified *K* = 2 as the optimal mode. At this level, the division of population genetic structure aligned with the basal clades of the phylogenetic tree, reflecting a pivotal historical divergence event in ancestral gene pools rather than corresponding directly to extant species diversity (Gilbert et al. 2012; Janes et al. 2017). At *K* = 4, the two putative new species formed distinct genetic lineages, while *Primula xingshanensis* and *P. rupestris* still remained undifferentiated, indicating greater divergence between the two putative new species. Only at *K* = 5 were all species resolved as independent lineages.

### 3.3 Morphological Observation and Comparison

Morphologically, the two putative new species are distinct from *Primula sinensis* and *P. rupestris* (with suborbicular and palmately lobed leaves) due to their oblong, pinnately lobed leaves. *P. xingshanensis* can also be distinguished from the two putative new species by its conspicuously decurrent, spathulate leaf base and a yellow throat with clear boundaries. Comparing the morphological differences between the two putative new species: *P. rongrong* is densely villous throughout the plant, has leaf lobes with sparse rounded teeth on the margins, and the basal pair of lobes is deeply divided to the petiole, degenerating into auriculate structures. Its corolla throat is light yellow without nectar guides. In contrast, *P. fujiangensis* is densely pubescent, has leaf lobes with numerous coarse teeth on the margins, a cordate base, and a white corolla throat with dark purple nectar guides, clearly distinguishing it from *P. rongrong*. The morphological comparison among these five related taxa is shown in Table 2.

**TABLE 2:** Morphological comparison of the new species with similar species.

Characters	*P. rongrong*	*P. fujiangensis*	*P. sinensis*	*P. rupestris*	*P. xingshanensis*
Stem	Multi‐branched	Multi‐branched	Few‐branched	Multi‐branched	Multi‐branched
Indumentum	Densely villous	Densely pubescent	Sparsely pubescent	Sparsely villous	Densely villous
Leaf shape	Oblong	Oblong	Ovate to subrotund	Ovate‐rotund to ovate‐elliptic	Bovate‐spatulate to spatulate
Leaf width	1–3 cm	1–3 cm	4–12 cm	2–5 cm	1–3 cm
Leaf lobing	Pinnately	Pinnately	Palmately	Palmately	Pinnately
Lobes	Sparse rounded teeth	Numerous coarse teeth	Numerous coarse teeth	Numerous rounded teeth	Entire margin
Leaf base shape	Cordate	Cordate	Cordate	Cordate	Cuneate
Leaf abaxial surface color	Green	Pale green, occasionally pale purple	Purplish‐red to pale purple	Pale green	Pale green
Flower color	Externally pink or light pink, internally white, centrally nearly white, light yellow or purple	Externally pink or light pink, centrally white	Externally purple, pink to white, centrally yellow	Externally pink or light pink, centrally yellow or white	Externally pink, centrally yellow
Nectar guides	Absent	Purple	Purple or yellow	Absent or yellow	Yellow

**FIGURE 5:**
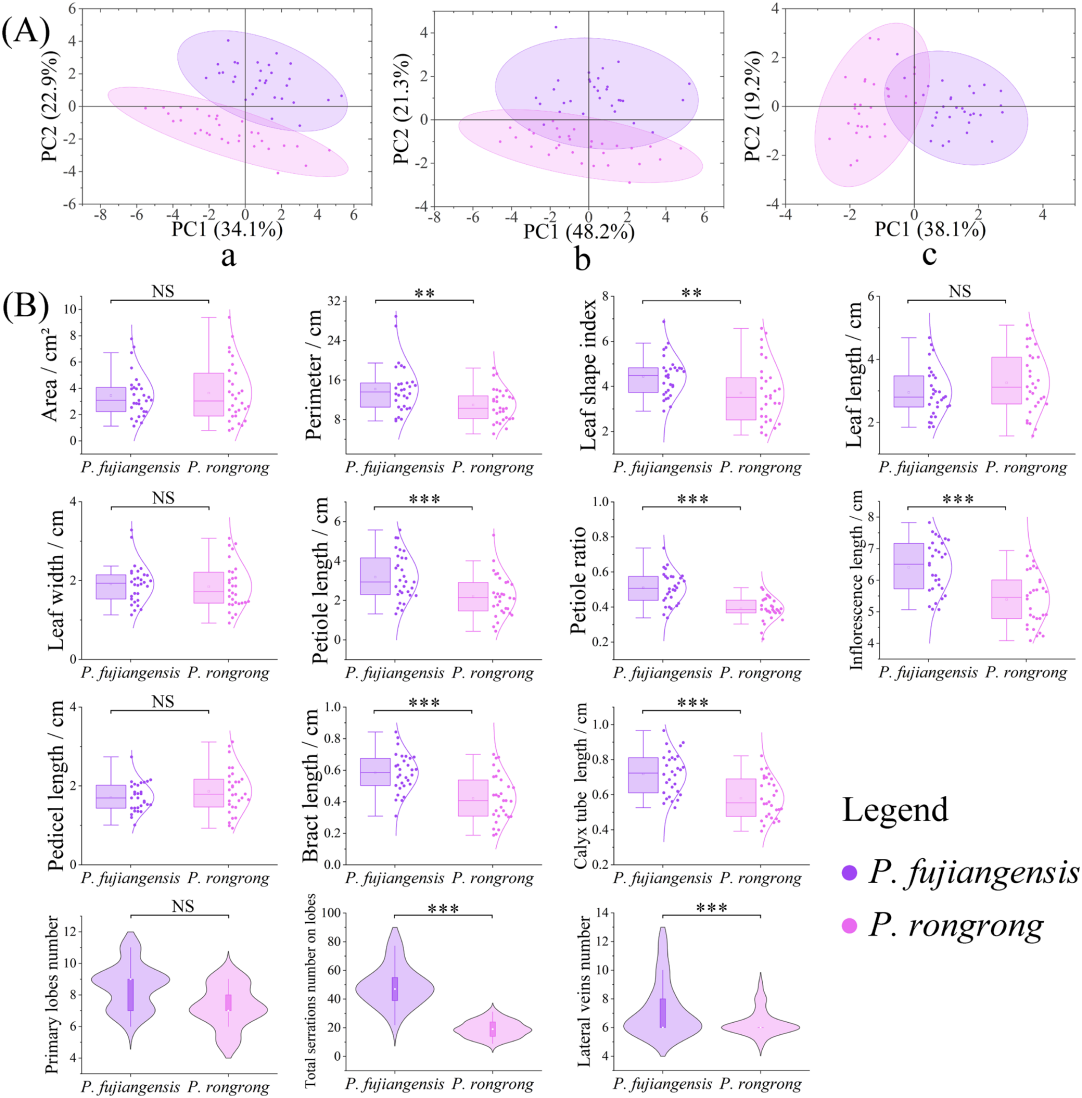
Morphological comparison between *Primula rongrong* and *P. fujiangensis*. (A) Principal component analysis (Aa) based on all 16 traits, (Ab) based on 10 leaf traits, (Ac) based on 6 floral traits. (B) Descriptive statistical analysis of 14 quantitative traits, in the intergroup comparisons, “NS” represents no significant difference (*p* ≥ 0.05), **p < 0.01, and ***p < 0.001.

PCA of the morphology of the two putative new species showed (Figure 5A) that the 95% confidence intervals of leaf and floral traits between the two groups exhibited some overlap. However, after incorporating all morphological indices, the overlap of confidence intervals narrowed, forming two distinct clusters with substantial differences. Significant difference analysis of 14 quantitative traits revealed that the two groups differed significantly in eight indices: perimeter, leaf shape index, petiole length, petiole ratio, bract length, calyx tube length, total serrations number on lobes, and lateral veins number (Figure 5B).

### 3.4 Fruit‐Setting Status of Artificial Pollination

The fruiting status statistics of artificial hybridization experiments are shown in Table 3. The average fruit set rate between heterostylous flowers of *Primula rongrong* was 80%, while that of *P. fujiangensis* was 67.5%, indicating that even with artificial pollination, there was a certain probability of abortion under our cultivation conditions. However, the fruit set rates of all interspecific combinations between *P. rongrong* and *P. fujiangensis* were at a low level, with an average of 13.75%, which was much lower than the intraspecific fruit set rates. This suggests that a certain degree of reproductive isolation exists between *P. rongrong* and *P. fujiangensis*.

**TABLE 3:** Fruiting status in artificial pollination experiments.

Combination	Number of fruit set	Fruit set rate (%)	Average fruit set rate (%)
R L ♂ × R S ♀	15	75	80
R S ♂ × R L ♀	17	85	
F L ♂ × F S ♀	13	65	67.5
F S ♂ × F L ♀	14	70	
R L ♂ × F S ♀	4	20	13.75
R S ♂ × F L ♀	3	15	
F L ♂ × R S ♀	1	5	
F S ♂ × R L ♀	3	15	

### 3.5 Species Delimitation between Two Putative New Species

In phylogenetic trees, the representation of monophyletic and paraphyletic clades provides an intuitive evolutionary framework for species delimitation, where monophyletic groups offer positive support for defining true evolutionary units (de Queiroz 2007; Carstens et al. 2013; He et al. 2022; Jiang et al. 2024). Typically, when describing a new species, its status as an independent taxon can be well evaluated if it forms a monophyletic clade in phylogeny and exhibits significant morphological differences from described sister clades. However, the two putative new taxa discovered in our study are sister clades, which precludes straightforward species delimitation based solely on clade relationships. To rigorously evaluate their taxonomic status, we employed morphological statistics, population genetic structure analysis, and hybridization experiments as auxiliary evidence for species delimitation. The results showed that the two taxa were easily distinguishable in morphological clustering, had significant differences in multiple morphological traits, exhibited a genetic distance greater than that between *Primula rupestris* and *P. xingshanensis* in sect. *Auganthus*, and displayed significant incompatibility in hybridization. Based on these findings, we conclude that both putative new taxa should be accepted as independent species, which are formally described herein.

## 4 Taxonmy

### 4.1 *Primula rongrong* S. Y. Zhang, Y. Hu & J. W. Shao, sp. nov. (Figure 6)

**FIGURE 6:**
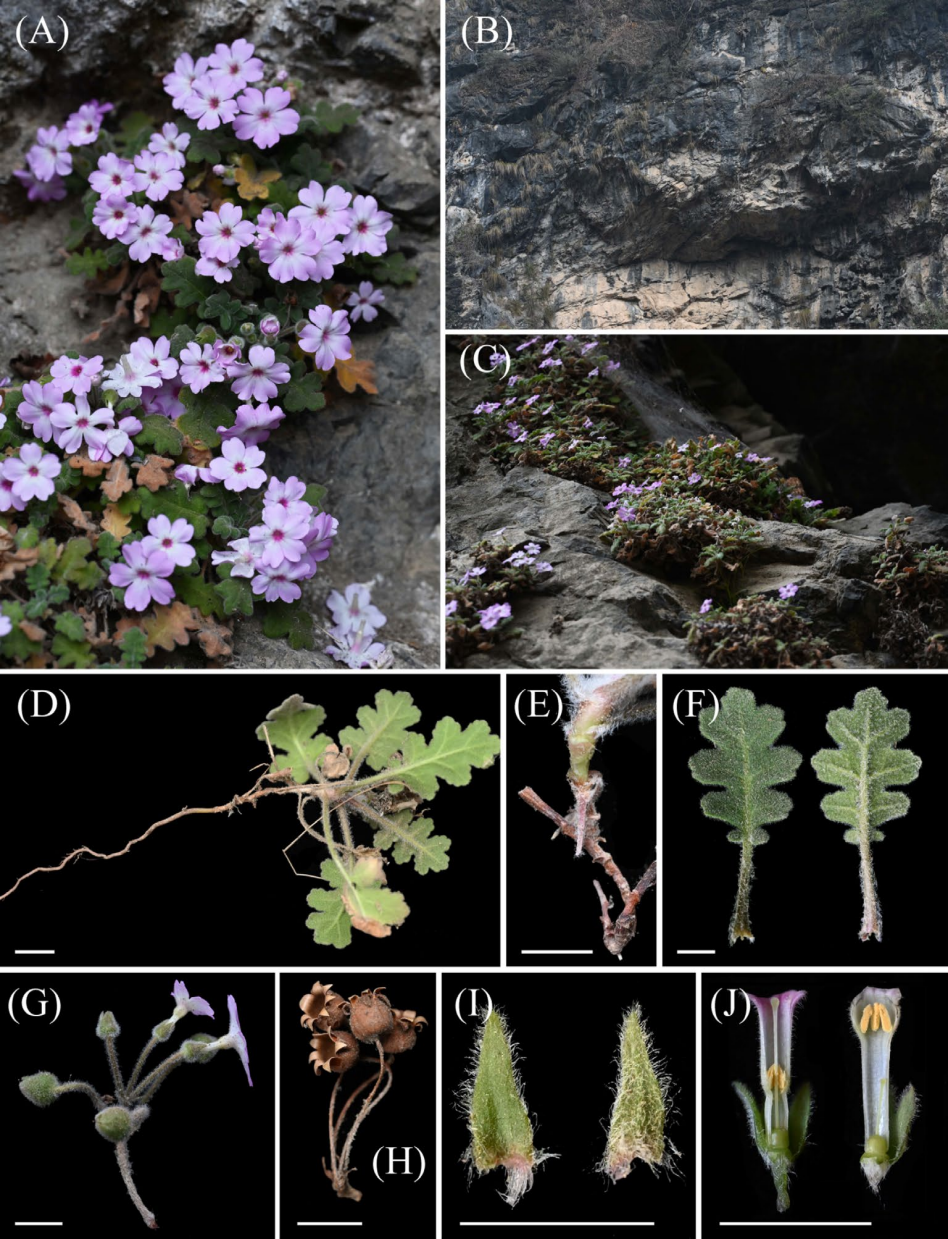
*Primula rongrong*. (A) Wild plants in the flowering stage. (B, C) Habitat. (D) Vegetative body, showing root system. (E) Stem. (F) Leaf, with the upper surface on the left and lower surface on the right. (G) Inflorescence. (H) Dry and dehiscent fruiting inflorescence. (I) Bract, with the upper surface on the left and lower surface on the right. (J) Dissection of flower tube, with long‐styled flower on the left and short‐styled flower on the right.

#### 4.1.1 Type

CHINA. Sichuan: Qingchuan County, Fangshi Town, 32°23′, 104°58′, elev. 913 m, 26 Feb 2024, in rock crevices of a cliff, Siyu Zhang & Yang Hu ZSY‐zbcz013 (holotype: ANUB 100111!; isotypes: ANUB 100112!, ANUB 100113!, ANUB 100114!, CSH 0219763!, NAS 00718036!).

#### 4.1.2 Diagnosis

*Primula rongrong* is overall similar to *P. xingshanensis* but is distinguished by the following features: leaf base cordate with auriculate lobes (vs. cuneate in *P. xingshanensis*), leaf lobes with sparse rounded teeth (vs. entire margin), and flowers lacking nectar guides and with indistinct light yellow or purple throats (vs. having yellow nectar guides and bright yellow throats).

#### 4.1.3 Description

Perennial herb, 5–10 cm tall, densely covered with white villous. Roots brown, fleshy, and brittle. Rhizomes brown, 3–15 cm long, branches above the middle (usually near the top), comparatively stout, with numerous dry leaf sheaths and old and young leaves at the apex. Leaves 7–15 in an open rosette; petioles 1.0–4.0 cm long, green, vaginate at base; leaf blades oblong, 1.5–6 × 1.3–2.5 cm, fleshy‐like, adaxially green to dark green, abaxially green, pinnately lobed to 1/2–2/3 of its width, lobes 7–9, the basal pair deeply lobed to the petiole, degenerated into auriculate shapes, other lobes rectangular, about 3 shallow lobes; cordate at base, lateral veins 2–3 pairs. Scapes 1–3 arising from each leaf rosette, 3–8 cm tall, umbels 1–2, superimposed, each umbel with 1–6 flowers; bracts lanceolate, 0.3–0.9 cm long, acute at apex, pedicels 1.5–3.0 cm long. Flowers heterostylous; calyx narrowly bell‐shaped, 6–9 mm long, 3–5 mm in diam., inflated at base in fruit, lobed to 1/3, lobes triangular, margin entire; corolla pink to nearly white, pubescent outside, throat purple, light yellow to nearly white, limb 2–2.5 cm in diam., lobes 5, broadly obovate, 2‐lobed at apex, parted to 1/5. Pin flowers with 10–12 mm tubes, stamens attached below the middle, and styles reaching or slightly protruding from the corolla throat, 9–11 mm long; thrum flowers with the same tube length, stamens near the corolla throat, style 4–5 mm long. Stigma globular; ovary globular, glabrous, ca. 2 mm in diam., ovules many. Capsule subglobose, 6–8 mm in diam., splits open at the top after ripe, lobes usually 7, triangular, curling outwards. Seeds many, ellipsoid, about 1 mm long.

#### 4.1.4 Phenology

Flowering in January to March, fruiting in May to June.

#### 4.1.5 Ecology and Geographical Distribution

It grows in the crevices of limestone cliffs along a river stretch approximately 2 km long; only one population has been found. This species usually grows together with *Clematis hastata* Franch. ex Finet & Gagnep., *Corydalis moupinensis* Franch., and *Corydalis edulis* Maxim.

#### 4.1.6 Etymology

The specific epithet of the new species is derived from the Romanized Pinyin of its Chinese name, which means hairy or furry, fitting the characteristic of the new species having hair all over the plants. Here, its Chinese name is proposed as “Róng róng bào chūn“ (绒绒报春).

#### 4.1.7 Conservation Status

This new species grows exclusively on rocks along a river valley approximately 2 km long, occupying an area of less than 10 km^2^. Although a narrowly distributed species, it has a large number of individuals and faces minimal human threats. Furthermore, the species is highly drought‐tolerant and exhibits exceptional environmental adaptability. Based on the IUCN Standards & Petitions Committee (2024) criteria and categories, and due to the absence of observed threats or population decline, we tentatively assess this species as least concern (LC).

### 4.2 *Primula fujiangensis* J. L. Gu, Ying F. Hu & S. Y. Zhang, sp. nov. (Figure 7)

**FIGURE 7:**
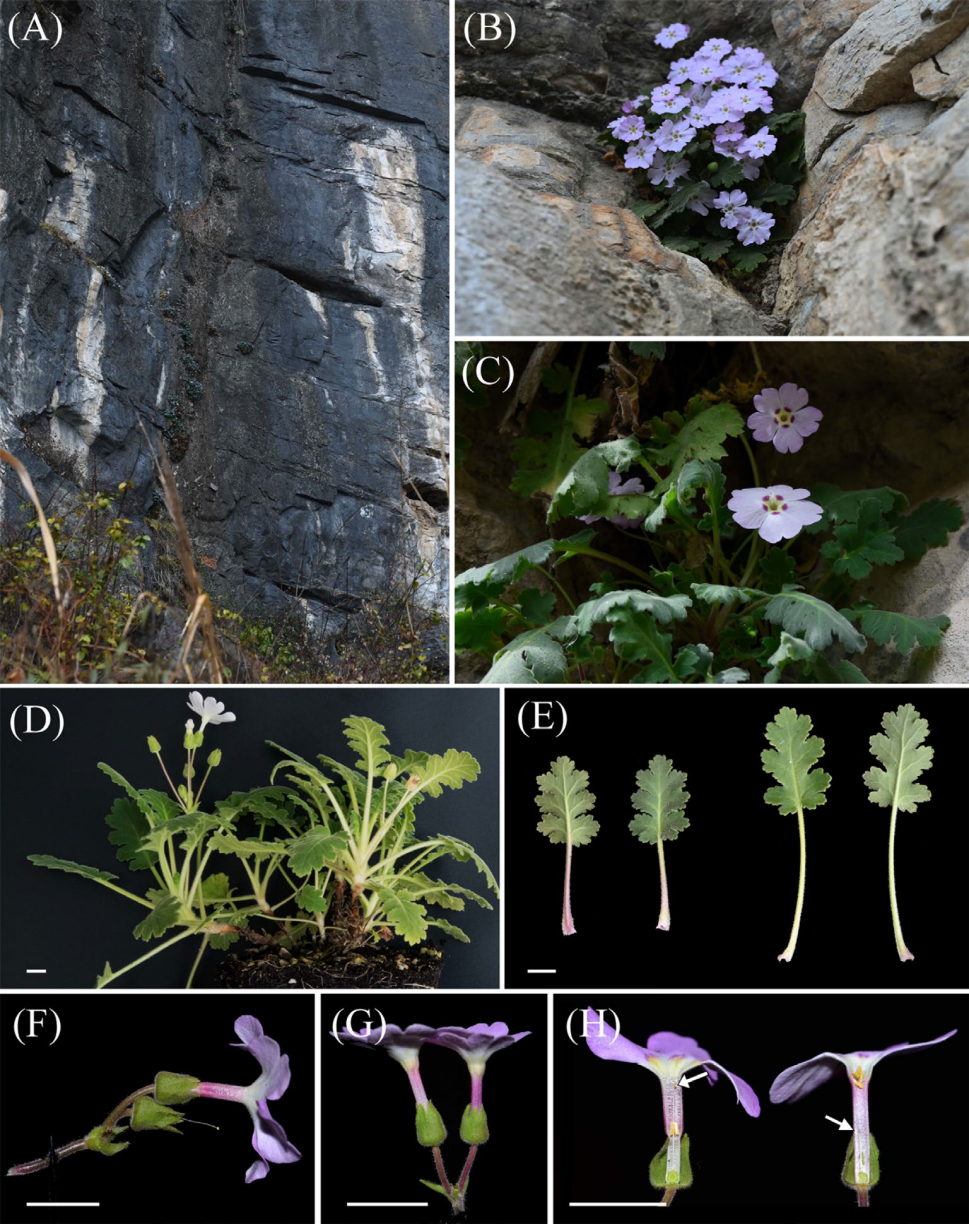
*Primula fujiangensis*. (A) Habitat. (B, C) Wild plants in flowering stage. (D) Lateral view of the plant, showing plant habit and stem morphology. (E) Leaves, with the middle two showing the upper surface and the two sides showing the lower surface. (F) Lateral view of the inflorescence. (G) Close‐up of a small flower from the side. (H) Dissection of the flower tube, with the long‐styled flower on the left and the short‐styled flower on the right. F, G, and H were photographed by Mr. Dechang Meng.

#### 4.2.1 Type

CHINA. Sichuan: Jiangyou County, Wudu Reservoir, 31°58′ N, 104°47′ E, elev. 1083 m, 27 Feb 2024, in rock crevices of a cliff, Siyu Zhang & Yang Hu ZSY‐zbcz008 (holotype: ANUB 100115!; isotypes: ANUB 100116!, CSH 0219762!, NAS 00718037!).

#### 4.2.2 Diagnosis

*Primula fujiangensis* is most similar to *P. rongrong* but can be distinguished by the following characters: plant entirely covered with short pubescence (vs. long pubescence in *P. rongrong*), leaf lobes with numerous coarse serrations on the margins (vs. sparse rounded serrations), leaf abaxial surface pale green or pale purple (vs. green), and flower throats white with distinct purple nectar guides (vs. light yellow or purple throats without nectar guides).

#### 4.2.3 Description

Perennial herb, 5–10 cm tall. Rhizomes brown, 3–10 cm long, branches above the middle (usually near the top), comparatively stout, with numerous dry leaf sheath and old and young leaves at the apex. Leaves 7–12 in an open rosette; petioles 2.0–8.0 cm long, green or red, vaginate at base, densely covered with white pubescence; leaf blades oblong, 3–6 × 1–3 cm, fleshy‐like, covered with short pubescence, adaxially green to yellow‐green, abaxially light green, pinnately lobed to 1/2–3/4 of its width, lobes 7–9(−11), rectangular, with 5–9 serrations; cordate at the base, and lateral veins 3–4 each side. Scapes 1–2 arising from each leaf rosette, 4–8 cm tall, covered with white pubescence; umbels only 1, with 2–6 flowers; bracts lanceolate, 0.5–0.7 cm long, acute at apex, covered with white pubescence, pedicels 1.5–3.0 cm long, covered with white hairs. Flowers heterostylous; calyx bell‐shaped, 6–8 mm long, 5–7 mm in diam., inflated at base in fruit, lobed to 1/3–1/2, lobes triangular, margin entire; corolla lavender, pink to nearly white, pubescent outside, throat usually white, rarely light yellow, often has purple nectar guides on the outer side, limb 2–2.8 cm in diam., lobes 5, broadly obovate, 2‐lobed at apex, parted to 1/6–1/3. Pin flowers with 11–13 mm tube, stamens attached below the middle, style reaching or slightly protruding from the corolla throat, 10–12 mm long; thrum flowers with 12–14 mm tube, stamens near the corolla throat, and styles 6–8 mm long. Stigma globular; ovary globular, glabrous, ca. 2 mm in diam., ovules many. Capsule subglobose, 6–8 mm in diam., splits open at the top after ripe, lobes usually 7, triangular. Seeds many, ellipsoid, about 1 mm long.

#### 4.2.4 Phenology

Flowering in January to March, fruiting in May to June.

#### 4.2.5 Ecology and Geographical Distribution

Growing in the crevices of cliffs in a dry limestone gorge, only one population has been found, intermingled with the critically endangered species *Urophysa rockii* O. E. Ulbr.

#### 4.2.6 Etymology

The specific epithet is derived from the native habitat of the new species, which is located beside the Fujiang River, the largest tributary on the right bank of the Jialing River (with a total length of approximately 700 km). Here, its Chinese name is proposed as “Fú jiāng bào chūn” (涪江报春).

#### 4.2.7 Conservation Status

This species is currently only found sparsely on a cliff with a unique habitat (extent of occurrence < 100 km^2^; area of occupancy < 10 km^2^). In recent years, this cliff has become a relatively well‐known rock climbing destination. Many bolts have been installed into the rock face, and climbers often step on the new species to gain better footing, resulting in its population visibly shrinking. Based on the IUCN Standards & Petitions Committee (2024) criteria and categories, we assess this species as critically endangered (CR B1ab + 2ab) to highlight the urgency of its conservation.

### 4.3 Key to Species of *Primula* Sect. *Auganthus*

**Table:** 

1	Leaf blade pinnately lobed	2
—	Leaf blade 5–9 deeply lobed	5
2	Plants terrestrial; perennial stem obscure; leaf blade pinnatisect	*P. filchnerae*
—	Plants petrophytic; perennial stem prominent; leaf blade pinnatifid	3
3	Leaf blade base cuneate, lobes entire	*P. xingshanensis*
—	Leaf base cordate, lobes serrulate	4
4	Entire plant villous; flowers without nectar guides	*P. rongrong*
—	Entire plant pubescent; flowers with purple nectar guides	*P. fujiangensis*
5	Stems few‐branched; leaves 4–12 cm wide, with coarse lobes, abaxially purplish red to pale purple	*P. sinensis*
—	Stems multi‐branched; leaves 2–5 cm wide, with crenate lobes, abaxially green	*P. rupestris*

## Author Contributions

**Si‐Yu Zhang:** investigation (equal), methodology (equal), visualization (lead), writing – original draft (lead). **Ying‐Feng Hu:** investigation (equal), methodology (equal), visualization (supporting), writing – original draft (equal), writing – review and editing (equal). **Jiu‐Lin Gu:** investigation (equal), methodology (supporting), visualization (supporting). **Yang Hu:** investigation (equal), methodology (equal), visualization (supporting). **Xiao‐Yu Si:** methodology (equal), writing – original draft (supporting). **Wei Zhang:** methodology (supporting), writing – review and editing (equal). **Jian‐Wen Shao:** conceptualization (lead), funding acquisition (lead), methodology (equal), writing – original draft (equal), writing – review and editing (lead).

## Conflicts of Interest

The authors declare no conflicts of interest.

## Data Availability

The chloroplast genome sequences used in this study have been deposited in NCBI; the accession numbers are shown in Table 1. The original morphological data and nuclear gene SNP locus matrix can be obtained from the following links: https://doi.org/10.5061/dryad.g79cnp62s.
